# Prescribing Assistive Technology for Cognition to Support Aging in Place: OTs’ Perspective

**DOI:** 10.1177/00084174251362524

**Published:** 2025-08-08

**Authors:** Amel Yaddaden, Julie Legault, Carolina Bottari, Quoc Dinh Nguyen, Nathalie Bier

**Keywords:** Older adults, Cognitive impairments, Assistive technology for cognition, Aging in place, Home safety, Déficiences cognitives, personnes âgées, sécurité à domicile, technologie d'assistance pour la cognition, vieillir chez soi

## Abstract

**Background.** With a rapidly aging population, ensuring the safety and independence of older adults, particularly those with cognitive impairments, is a key public health priority. Occupational therapists (OTs) play a crucial role by recommending assistive technologies for cognition (ATCs) to support this population. However, little is known about how OTs choose ATCs, and the rehabilitation strategies involved in their implementation. **Purpose.** This study examines OTs’ perspectives on prescribing ATCs to support aging in place, focusing on (1) factors influencing ATC recommendations and (2) effective rehabilitation strategies. **Methods.** We conducted a descriptive qualitative study with 15 geriatric-focused OTs across three focus groups. Discussions were analyzed through three steps: coding, refining, and creating data matrices. **Findings.** OT recommendations are influenced by client factors (e.g., learning ability), specific tasks (e.g., medication management), and contextual elements (e.g., financial support). OTs employ cognitive rehabilitation, practice simulations, and caregiver collaboration strategies to support ATC integration. **Conclusions.** Understanding how OTs choose and apply ATCs provides insights to optimize their use in geriatric care, promoting safer, independent living for older adults.

## Résumé

**Description.** Compte tenu du vieillissement rapide de la population, assurer la sécurité et l'autonomie des personnes âgées, en particulier celles qui ont des déficiences cognitives, est une priorité clé en santé publique. Les ergothérapeutes jouent un rôle crucial en recommandant des technologies d'assistance pour la cognition (TAC) pour cette population. Cependant, on sait peu de choses sur la façon dont les ergothérapeutes choisissent les TAC, et sur les stratégies de réadaptation impliquées dans leur utilisation. **But**. Cette étude examine les perspectives d'ergothérapeutes sur la prescription de TAC pour aider à vieillir chez soi, en se concentrant sur (1) les facteurs influençant les recommandations en matière de TAC et (2) les stratégies de réadaptation efficaces. **Méthodologie.** Nous avons mené une étude qualitative descriptive auprès de 15 ergothérapeutes travaillant en gériatrie et dans trois groupes de discussion. Les discussions ont été analysées en trois étapes : codage, raffinement et création de matrices de données. **Résultats.** Les recommandations des ergothérapeutes sont influencées par des facteurs liés aux personnes clientes (p. ex., capacité d'apprentissage), aux tâches à accomplir (p. ex., gestion des médicaments) et au contexte (p. ex., soutien financier). Les ergothérapeutes ont recours à la réadaptation cognitive, aux simulations et aux stratégies de collaboration avec les proches aidants pour aider à l'intégration des TAC. **Conclusion.** Comprendre comment les ergothérapeutes choisissent et appliquent les TAC fournit des informations pour optimiser leur utilisation dans les soins gériatriques, favorisant ainsi une vie plus sécuritaire et autonome pour les personnes âgées.

## Introduction

Safety in home support is a complex, multidimensional issue closely tied to functional independence and a person's ability to safely perform daily tasks (De Courval et al., 2006). Occupational therapists (OTs) assess safety risks in older adults’ homes, focusing on high-risk activities like cooking, nighttime outings, and medication management (Bourgeois et al., 2009; De Courval et al., 2006). Cognitive impairments, especially from conditions like Alzheimer's disease, increase the likelihood of risk-taking behaviors, heightening these safety concerns (Bourgeois et al., 2009; De Courval et al., 2006; Giebel et al., 2015).

To manage risk-taking behaviors, OTs can introduce assistive technologies for cognition (ATC) (Aboujaoudé et al., 2021; Thawisuk et al., 2023). For example, they can implement the use of automatic reminder systems via digital applications (e.g., digital clocks) or recommend the integration of an automatic pill dispenser (Thawisuk et al., 2023). Automatic pill dispensers can assist users by alerting them when it is time to take their medication and monitoring actual intake through various sensors (Patel et al., 2022). Over the past 15 years, several research initiatives have been undertaken in the field of ATCs (Ienca et al., 2017) and show that: 1) older adults tend to be very interested in these technologies (Peek et al., 2014), 2) they can learn to use them with appropriate rehabilitation interventions such as cognitive strategies, scenario simulations and learning techniques (Imbeault et al., 2018a; Imbeault et al., 2018b), and 3) over 50% of OTs questioned in a nationwide survey are aware of technological aids for older adults, particularly those providing cognitive support (Aboujaoudé et al., 2021). Moreover, numerous products designed specifically for older adults to support various daily activities have recently emerged on the market. Thus, the current context is very favorable to the use of these technological aids in clinical practice (Sixsmith et al., 2020). However, the reasons that underlie OTs’ decisions to integrate ATCs into their clinical practice are still poorly understood. Research shows that OTs do not recommend them to just any client, especially when the latter are older adults, sometimes out of fear that the person will simply not be able to use them (Aboujaoudé et al., 2021). Understanding the factors influencing OTs’ decisions could answer: (1) **What** person-, activity-, technology- and context-related conditions lead OTs to recommend cognitive technological aids? and (2) **How** do OTs support the learning and use of these aids? Exploring these questions is essential to understanding the adoption and integration of technological aids in OT practice with older adults.

### Guiding Conceptual Framework

In OT, technological aids supporting cognition are categorized under “assistive technology” (AT), which is defined as health technologies designed to enhance functioning and promote participation in meaningful roles (World Health Organization, 2024). These technologies can reduce dependence on healthcare services and human assistance, fostering greater independence. According to Gitlin (2002), introducing AT into a person's daily routine involves multiple interventions such as modifying their environment, behavioral changes and activity adjustments for effective use. This necessitates an in-depth analysis of the person, the technology, the activity, and the context to ensure proper integration. The Human Activity Assistive Technology (HAAT) model is an used analytical framework in OT (Cook & Polgar, 2015; Giesbrecht, 2013). It represents human conditions (H = human), activity (A = activity), and technology (AT = assistive technology) as an interconnected system. In this model, AT acts as a facilitator, supporting specific human functions (e.g., abilities, experiences, or motivation) and tasks within an activity (e.g., medication management). Its effectiveness depends on the context, such as the physical, social, cultural, or institutional environment in which the activity occurs.

## Study Objectives

This study aims to explore the perspective of OTs working with older adults with cognitive impairments regarding the reasons that lead them to recommend and implement technological aids to support cognition. More specifically, we aim to identify (1) the factors related to the person, the activity to be supported, the technology itself, and the context in which it is used, that lead OTs to recommend ATCs (rehabilitation strategies) and (2) the interventions implemented for the effective use of technological aids.

## Methodology

### Study Design

This study follows a qualitative descriptive design. Researchers conducting this type of study attempt to stay close to their data, the words used, and events described to ensure the validity of the data (Paillé, 1996). A descriptive approach was employed to document the phenomenon under investigation—the prescription of ATCs by OTs. To gather in-depth insights, data were collected through focus groups, allowing participants to share their experiences and perspectives on the prescribing process (Krueger, 2014; Winke, 2017). The lead author is an occupational therapist and researcher with expertise in technologies for aging in place. Their research focuses on the role of evidence-based practices in occupational therapy and the impact of technologies on social participation for older adults with cognitive impairments. Adopting a post-positivist epistemological stance, they prioritize a rigorous methodological approach while ensuring fidelity to participants’ experiences and perspectives (Ryan, 2006).

### Participants

To be included in the study, potential participants had to (a) have national OT license, (ii) have a minimum of one year of experience in geriatric care, and (iii) willing to participate in this study*.*

### Ethics

The Research Ethics Board of the Aging-Neuroimaging Research Ethics Committee of the *CIUSSS du Centre-Sud-de-l’île-de-Montréal* approved the project (#2024-1960, CER VN 23-24-18*).* The participants provided written and informed consent to participate in the study. This study was conducted following ethical principles for research involving human participants, ensuring voluntary participation, confidentiality, and the right to withdraw at any time.

### Data Collection

Participants were recruited through announcements posted on virtual occupational therapy groups on social media networks. Participants were divided into groups of approximately 4 to 6 people, forming 3 focus groups until data saturation was reached (Chenail, 2016). Saturation is important for guaranteeing the validity and reliability of the results of a qualitative study. It indicates that the phenomenon studied has been thoroughly explored and that the conclusions drawn are based on sufficiently rich and diversified data (Hancock et al., 2016). Participants took part in one or two sessions, each lasting about an hour. These sessions were conducted either virtually or in person depending on participants’ availability. A **semi-structured interview guide** (see Table 1), approved by the Ethical review board guided the discussions throughout the focus groups.

**Table 1.** Focus Group Interview Guide.

Overall, what do you believe are the key conditions (characteristics of the older adult with cognitive impairments, the activity they perform, and their living environment) that lead you to recommend an assistive technology for cognition for a particular person? – What makes you hesitant to recommend assistive technology for cognition? – Under what circumstances is the intervention a success versus a failure? When it comes time to recommend assistive technology for cognition in your clinical practice, what are the characteristics of the company that offers ATCs that lead you to recommend one over another (for two equivalent assistive technologies)? – What information about the product increases or decreases the likelihood that you will recommend an assistive technology for cognition? – Name essential criteria that you consider for recommending ATCs from one company rather than another What interventions and strategies, in your opinion, could support the learning and use of assistive technology for cognition?

A moderator (A.Y.) was responsible for asking questions and guiding the discussions. An assistant moderator (J.L.) was responsible for taking notes and validating the discussion content with the group at the end of each topic. All group interviews were recorded to facilitate data analysis. Transcriptions of the audio recordings were made using a secure transcription application.

### Data Analysis

We used a content analysis process that involves four key steps: (1) preparing the material by defining objectives and establishing the corpus, (2) conducting an initial exploratory reading, (3) coding using predefined and emerging categories, and (4) analyzing the data to synthesize findings and draw conclusions(Intissar & Rabeb, 2015). The primary author (A.Y.), a researcher specializing in qualitative analysis, manually conducted a semi-deductive content analysis method, which applies a structured coding system to develop clear, coherent, and relevant categories that highlight the specific characteristics of the studied material (Intissar & Rabeb, 2015; Paillé, 1996). Coding aimed to assign labels (codes) to relevant units of meaning from the corpus, such as words, phrases, or paragraphs. She relied on the predefined categories of the HAAT conceptual framework (Cook & Polgar, 2008). The first list of codes was fully co-validated independently by a co-researcher (J.L.) and a senior qualitative researcher (N.B.) to reach a consensus. Then, for each category, sub-themes were regrouped inductively from the codes. Once coding was complete, matrices were developed to reduce the set of codes to a more manageable and easier-to-conceptualize format. To validate the analysis process and ensure rigor, a senior researcher specialized in qualitative analysis (N.B.) also co-validated the second-level matrices.

## Results

A total of 15 OTs took part in the study, including 12 women and 3 men. Their years of experience ranged from 1 to 32 years in diverse practice settings such as home support (n = 5), outpatient clinics (n = 1), intensive functional rehabilitation units (IFRU) (n = 5), post-acute care (n = 2), and day centers (n = 1). Their perceived use of ATCs in their practice ranged from little to quite a lot, with only one participant reporting extensive use. Table 2 shows the characteristics of the participants in each of the focus groups.

**Table 2.** Sociodemographic Characteristics of Participants.

# Group & Participant	Gender	Clinical Setting	Years of Experience	Perceived use of ATCs in Practice
Group 1	F	Home Support	18 years	Little
	F	Outpatient Clinic	14 years	Little
	F	Home Support	3.5 years	Moderate
	F	Home Support	5 years	Little
	F	Home Support	2 years	Moderate
Group 2	M	IFRU	5 years	Moderate
	F	Post-acute	7 years	Little
	F	IFRU	1 years	Little
	F	CPA	11 years	Little
	M	Day Center	12 years	A lot
Group 3	F	IFRU	3 years	Little
	M	Home Support	15 years	Moderate
	F	Post-acute	32 years	Little
	F	IFRU	32 years	Little
	F	IFRU	17 years	Moderate

Note. IFRU: Intensive Functional Rehabilitation Unit; CPA: Early Assisted Discharge.

### Key Conditions

**Figure 1.**
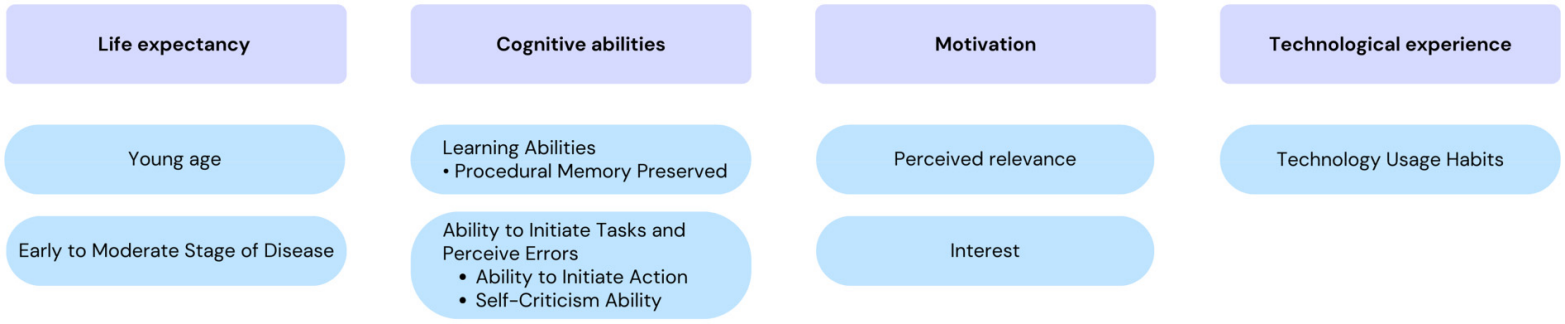
Human-Related Conditions That Lead OTs to Recommend an ATCs.

**Figure 2.**
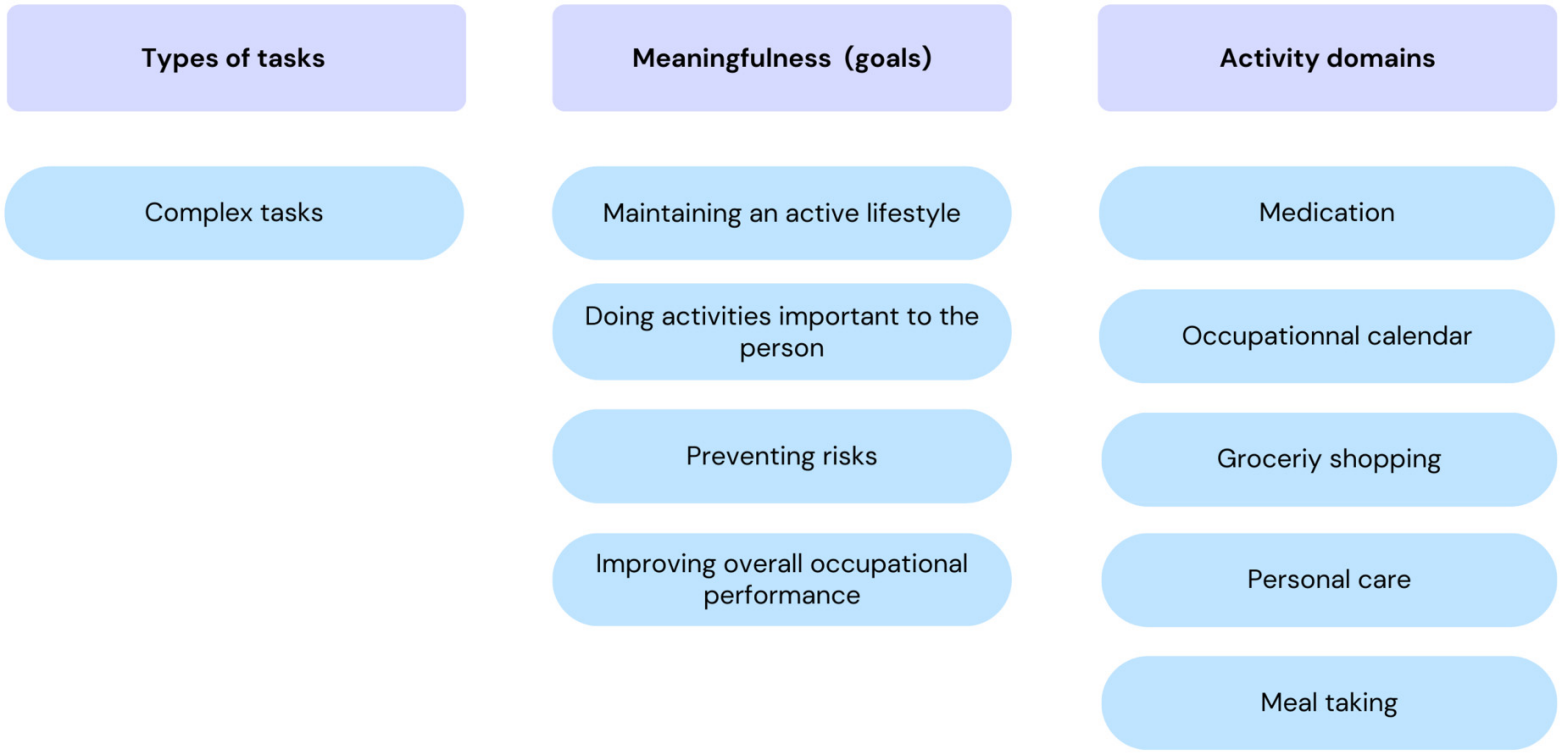
Activity-Related Conditions That Lead OTs to Recommend an ATC.

**Figure 3.**
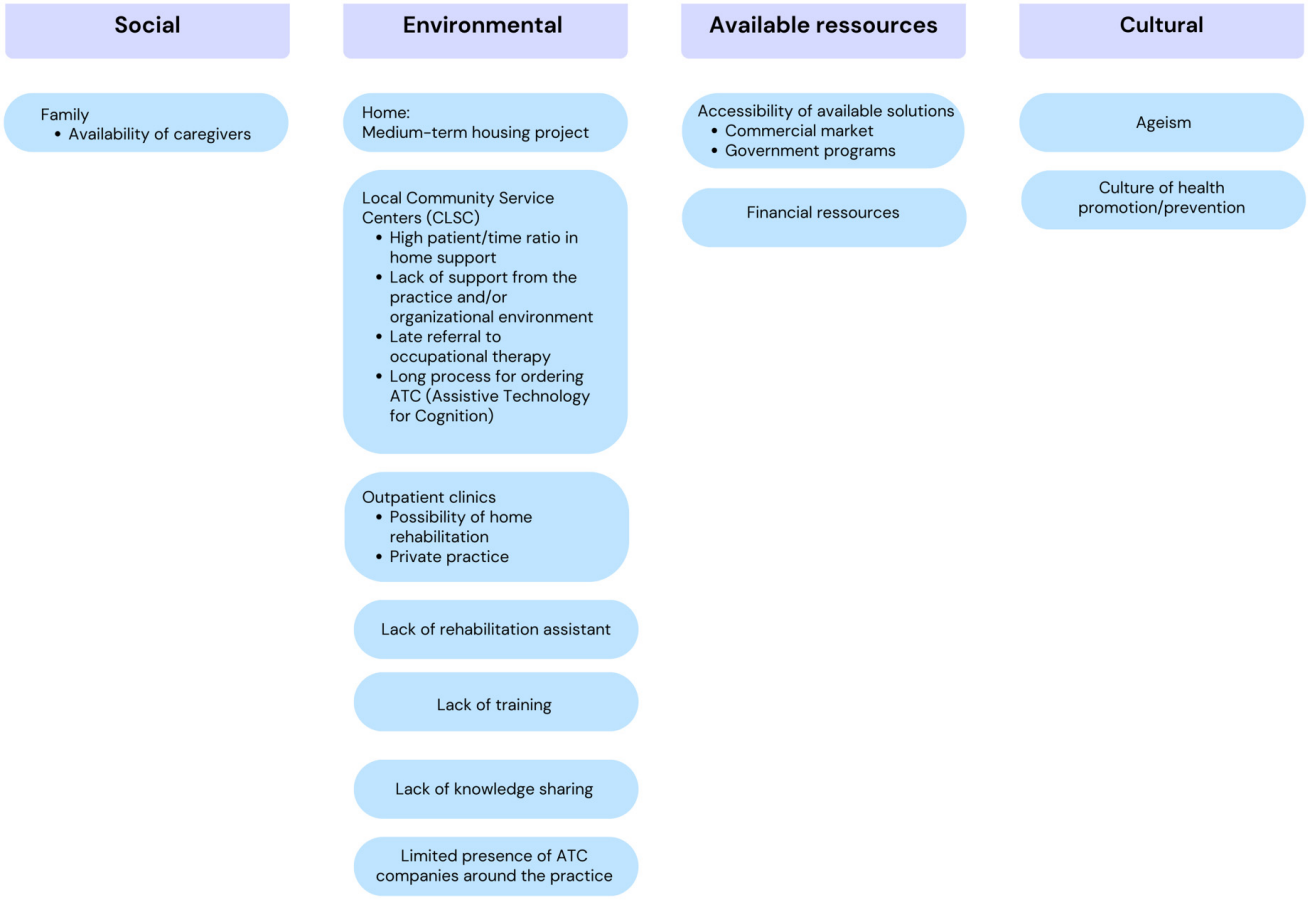
Contextual Conditions That Lead OTs to Recommend an ATC.

Our first objective was to identify the key conditions that lead OTs to recommend ATCs. According to the HAAT model, these are divided into human conditions, the activity to be supported, the technology itself and the context in which it takes place (see Figures 1, 2 and 3).

### Human-Related Conditions

Participants highlighted 4 key human dimensions influencing OTs’ recommendations for technological aids to cognition: life expectancy, cognitive abilities, motivation and technological experience. Figure 1 summarizes these factors.

For older adults aged 65–80, life expectancy is considered as a favorable factor due to their potential to adapt to and benefit from new technologies. Conversely, those in older age brackets (90+) may lack sufficient time to integrate these technologies meaningfully, potentially reducing intervention interest and effectiveness. About this, a participant said:

“…We have a very diverse clientele—some are 72, others are 99. With the younger ones, who often already use a cellphone, it's easier to explore new technologies. But for the older ones, it's not always worth introducing something new…” (FG2 – P2, #12)

This also applies to disease severity, as participants noted that advanced stages lower the likelihood of benefiting from such interventions. Regarding cognitive abilities, OTs highlighted learning abilities, particularly the preservation of procedural memory, as crucial when prescribing ATCs, as it supports the acquisition of automatisms or routines essential for learning ATC use. On this subject, one participant said:

“For functioning […], there are certain types of major neurocognitive disorders which no longer have **procedural memory**. I'm thinking, for example, of **Parkinson's disease**, where it's going to be difficult to even use that **type of memory** to develop new patterns of automatic behaviours to learn something new … But in **Alzheimer's disease, for example, in which it is preserved for quite a long time and is procedural, we could try**” (FG1 – P2, #118).

Another factor related to cognitive abilities highlighted by the participants is task initiation and error perception. For older adults who struggle with initiating actions, ATC reminders are a preferred intervention, as home visits are costly and limited. Also, OTs note that it can be challenging to encourage ATC use in older adults who lack self-evaluation capacity, as they must recognize their limitations to accept assistance. As one participant put it:

“I see it as a **lack of judgment and self-assessment ability** […] you know […] we won't even get to the stage of implementing technological aids because we won't even be able to evaluate […] that's an obstacle I've noticed” (FG3 - P3, #226).

Another human dimension to consider is the person's motivation which, according to OTs, comes down to the person's interest in using new technologies and their perceived usefulness to them. On this subject, one participant said:

“Then in the interests… I would also add the **perceived usefulness** by the patient, he must be convinced that it can help them” (FG2 - P3 – #126).

Lastly, technological experience is an important dimension and one that relates mainly to a person's technology usage habits. Having used technology before and being able to use it is a prerequisite that is considered and influences OTs’ decisions to prescribe an ATC. One participant explained how clients’ familiarity with technology and available devices influenced what could realistically be implemented:

“*If they already have habits or a device, you know, you can try telerehabilitation, but if there's no computer… With their tablets, cellphones, computers—what do they have, what do they use?… I had clients still using flip phones, so that limits what we can do. There are some prerequisites*….” (FG2, P5 – #78)

### Activity-Related Conditions

Participants highlighted three activity characteristics that influence OTs’ ATCs recommendations: type of task, meaningfulness, and activity domains. Figure 2 summarizes these elements.

In terms of the type of task, OTs were unanimous: they prescribe ATCs for so-called “complex” activities. These include activities with several steps and many components to consider, i.e., planning and organization, tasks that the person does infrequently, that are not automatic, and that require more decision-making. According to OTs, ATCs are used to alleviate the complexity of these tasks. As one participant put it:

“Well, you know, **a complex activity…** **there are more steps, there are more components** to take into account. You know, tasks, you know, that maybe we're less used to doing, but that isn't, you know, automatic, no automatic behaviours, so you know, what they require is to **make more decisions** that have to support things, so that [ATCs] can come to simplify some of those aspects, but that would be where I'd recommend [ATCs] more…” (FG2 - P4 - #220).

In terms of meaning, OTs will use ATCs as an intervention when they meet one or more of the following goals: (1) to maintain an active lifestyle, (2) to perform activities important to the person, (3) to prevent safety risks, and (4) to improve the person's overall occupational performance. Thus, according to OTs, the use of an ATC should not “triple” the time needed to perform an activity. The use of an ATC must improve not only safety, but also the person's functioning, and free up time for other activities. On this subject, one participant said:

“[…] Well technologies, technical aids will enable the person to **work less hard, but to do more things during the day**. […] So that they can also do other things in their daily life […]” (FG2-P3 #28)

### Context-Related Conditions

According to our results, four contexts encourage OTs to recommend ATCs: (1) social, (2) environmental, (3) financial, and (4) cultural. Figure 3 summarizes these factors.

The social context primarily refers to the availability of family caregivers. This was identified as a key condition for prescribing ATCs, because, according to the participants, the presence of a relative enables the practice of using ATCs with the person and ensures that it works well. A relative can also monitor and support learning, which encourages OTs to consider this type of intervention. On this subject, one participant said:

“Well, it's clear to me that it weighs in the balance. Are there caregivers, are there family caregivers, who can be present, who can practice with the person using this technical aid to make sure it's working properly?” (FG1, P4 - #120).

Regarding the environmental context in which OTs are likely to prescribe ATCs, several factors related to the home and the clinic were discussed. For home, the medium-term plans, particularly any change in the living environment anticipated in one year, are a crucial factor to consider. One participant said:

Then that's it, I also see it in terms of the family's or the person's medium- to long-term forecasts about their plans. Of course, you know if the person is planning to move to a long-term care home in 6 months, are we going to invest the time to train the person with a new technology? So, there's also all that [OT practice contexts] in-home support or private practice that will influence (FG1, P5 - #248).

At the clinical level, clinicians in homecare service centers highlight challenges in using ATCs as an intervention tool for OTs. Referrals to specialist care, such as OT, often occur only once issues are advanced, resulting in OTs being called in too late, with limited rehabilitation potential and minimal opportunity to implement ATCs in optimal conditions. Participants noted that many individuals on waiting lists could benefit from ATCs, but by the time they access OT services, it is often too late to intervene effectively. As one participant put it:

“There are a lot of elements related to the **practice context** that means that even if the situation would be ideal for implementing technology, we don't necessarily have the chance to put it in place […] but I think that in homecare when we're referred for neurocognitive care, we're often referred when the fire has already started**. It's rare to catch people early on, when they have rehabilitation potential,** you know, for a learning approach and all that. We're often referred to when there's a risk identified either by a relative or by the doctor or by the team. So, we're in fire extinguishing mode […] “ (FG1, P6 - #192).

On the other hand, the outpatient settings offer opportunities for home-based rehabilitation and private practices that facilitate the recommendation of ATCs by OTs. In outpatient clinics, the OTs transmit their recommendations directly to the persons and assess whether they are proactive and implement the advice given. On this subject, one participant said:

“That the outpatient intervention context perhaps facilitates the use of these technologies [ATCs] versus inpatient” (FG1, P5 - #62).

&

“[…] it's where externally with the pain clinic or when I transmit my recommendations, I can do so directly with the patient and that the person will be proactive and will implement it” (FG2, P3 - #98).

In the practice context of geriatric OTs, participants reported limited advanced knowledge of cognitive disorders and ATC use, citing a lack of training and peer knowledge-sharing. Limited interaction with ATC companies may contribute to this knowledge gap. OTs’ familiarity with technology directly influences their ability to prescribe ATCs; for instance, when a colleague returns from a symposium on ATC solutions, OTs are more likely to recommend these technologies. On this subject, one participant said:

“There's also a lack of training, I think, which greatly limits the use of technological aids… so if we were better trained in this area, we'd be more inclined to use them… so these are two elements that I think are big obstacles to the use of technological aids at the organizational level…” (FG1, P3 - #158).

In terms of available resources, the accessibility of solutions is influenced by the commercial availability of the technologies and government programs that offer them. Financial limitations, according to participants, lead OTs to recommend technologies that the person already has at home. They will avoid suggesting the purchase of new technologies and prefer something that is already accessible to the person which they will encourage to use or adapt according to the person's environment and abilities. As one occupational therapist put it:

“You know in-home support we always think about (the person's) home, you know, where everything is as close to home as possible… that we don't need to ask the person to buy something new that you know is already accessible, we use our imagination to make it very simple. […] So, it takes something accessible” (FG1, P3 - #128).

Finally, in terms of cultural context, two main factors emerged from discussions with OTs. First, being immersed in a culture of health promotion/prevention helps promote the use of ATCs. However, the presence of ageism in society hinders the use of this intervention modality. According to OTs, even older adults themselves can be ageist, limiting their acceptance of the intervention. As one participant put it:

“I have to admit that we're very well placed in OT to remove this stigma of ageism because ageism exists in society, and then even the older adults themselves, they do self-ageism by saying that ‘'ah no, forget it, I'm 85, I'll never be able to use it'” (FG3, P3- #266).

### Technology-Related Conditions

**Figure 4.**
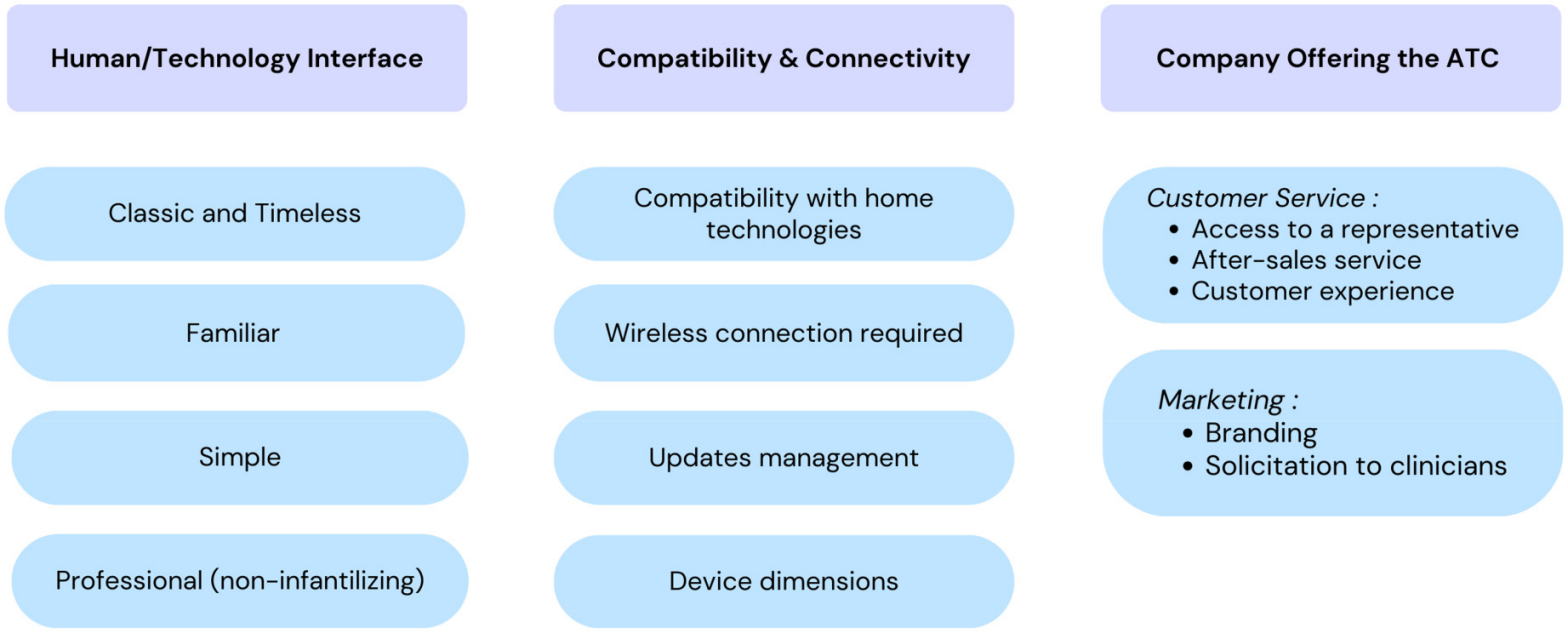
Technology-Related Conditions That Lead OTs to Recommend an ATC.

The technological conditions influencing OTs’ recommendation of ATCs can be divided into several key aspects, including (1) the human-technology interface, (2) compatibility and connectivity, and (3) the companies providing these technologies. Figure 4 summarizes these factors.

The human-technology interface is fundamental, as it guides OTs in prescribing ATCs for older adults with reduced dependence. Participants indicated that an ATC should have a classic, familiar, simple, and professional interface, resembling everyday objects rather than appearing overly technological. Occupational therapists note that older adults are often deterred by high-tech solutions, which supports recommending more traditional and familiar alternatives. On this subject, one participant mentions:

“One of the things that isn't always easy for customers, […] it's the little icons that look very easy when you have the back arrow that says go back, well it's not, it's not instinctive. For someone who doesn't use a computer or a cell phone, the little arrow at the bottom that's incomplete or the square that means reopen all windows, so something that's much more familiar than icons” (FG3, P6 - 412).

OTs also highlighted factors related to compatibility and connectivity, such as compatibility with technologies already present in the home, the importance of verifying whether the product requires a Wi-Fi connection, managing updates and device dimensions. It is crucial to check whether devices require manual updates, as the person may lose control of their ATCs. Application updates are often difficult to manage for this clientele.

Customer service and marketing by ATC providers are key factors that influence OTs’ recommendations. Impeccable service—including access to a representative, after-sales support, and a positive customer experience—facilitates ATC adoption. OTs value having a representative visit the clinic to present ATCs, answer questions, and provide demonstrations to support clinical integration. A weekend helpline, accessible support for technical issues, backup plans for device failures, and long-term follow-up help reduce OTs’ workload and increase recommendation likelihood. Customer service must address both clinical and client realities. Regarding customer service, one participant said:

“We asked the representative to have a reliable line so that people could call on weekends when there's a problem because it's a misery on Saturdays and Sundays (when we're not available to answer calls) […] It's like, you're the one who prescribes it, you're the one who must put in the work. […] But it's just that it adds so much responsibility (on our shoulders). You know, we're already so swamped with waiting lists, and then seeing everyone, that if on top of that, we must deal with technological bugs, which are very frequent in some companies, it's too much of a burden…” (FG1, P5 - #262).

In addition, quality marketing, including strong branding and solicitation of clinicians, would contribute to the widespread use of ATCs by OTs. Indeed, OTs have observed that some companies optimize their visibility to such an extent that they are predominantly in the minds of clinicians when it comes to recommending an ATC. Company awareness and the widespread use of the company's proposed ATCs by other clinicians also play a significant role. Regarding branding, one participant said:

“I come back to the positive… […] let's say (name of a fall detector company), they bombard me with documents, I get like emails all the time, coupons, all that stuff… it makes me remember it. I carry them with me all the time, so when my clients ask me for, say, a fall detection system, they're the first ones to come to mind, even though I know there are others (because they're readily available)” (FG1, P4 - #196)

In summary, if OTs are to recommend ATCs, it is essential on the technological side that they are supported by good customer service and accompanied by targeted marketing efforts to maximize their adoption and continued use.

### Interventions and Strategies

**Figure 5.**
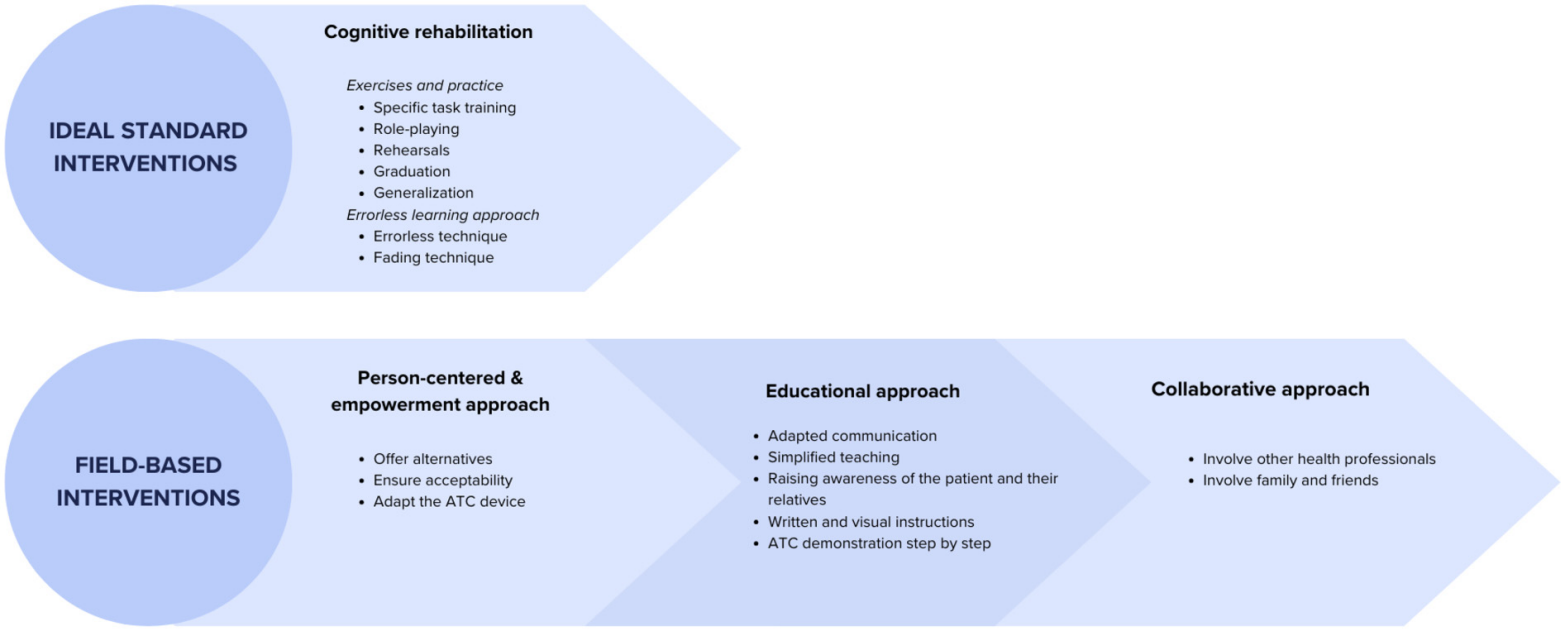
OTs Comprehensive Interventions to Support Learning & Effective Use of ATCs.

Our second objective was to identify the interventions implemented by OTs to ensure the effective use of ATCs by their clients. Four different **standard OT intervention approaches:** 1) cognitive rehabilitation, 2) person-centered & empowerment approach, 3) educational approach and 4) collaborative approach. Figure 5 summarizes these approaches.

As the most effective and **ideal interventions** used to ensure the successful use of ATCs by their clients, OTs reported several learning techniques and strategies they use, such as practice trials, role-playing, repetition, activity gradation and generalization. These are part of the *cognitive rehabilitation approach* and enable the practice of using ATCs in realistic contexts by doing or simulating tasks of daily living, thus promoting the achievement of daily activities with ATCs. They require several sessions over an extended period, to help integrate ATCs into the person's routine. Recognized cognitive rehabilitation methods, such as the errorless method and fading, are also used to facilitate the learning of specific tasks using ATCs. On this subject, one participant said:

“For technological aids to be integrated into a routine, there has to be a certain learning curve, it has to be errorless, it has to be guided, you know it takes a certain amount of time” (FG1, P2 - #118)

&

“[…] Errorless learning, the principles of cognitive rehabilitation… The other thing is repetition. It's often said that what will help this learning technique is to do it repeatedly, but we don't always have the resources or the time” (FG1, P3 - #123).

According to OTs, when they can use specialized learning techniques, such as *fading techniques*, to teach older adults how to use cognitive assistive technology, it helps establish a habit in the person, making the use of the technology routine. About this, a participant said:

“[…] I think it can be in the establishment of the habit. When the older person can continue learning the use of the ATC. Because when we can **fade it** […], they develop the habit, and it becomes a routine that has been well established and implemented.” (FG2, P5 – #86)

The second intervention category centers on a person-centered and empowerment approach, emphasizing the need to offer multiple ATC options to older adults. It is essential to assess the acceptability of these alternatives based on the individual's specific skills before prescribing. The occupational therapist can then tailor the ATCs to the older adult's abilities and needs, facilitating adoption and effective use. On this subject, one participant said:

“What I would add is that I have a concrete example with a patient whose music is on her Bluetooth device, so we adapted the iPad to make it easy to use. With the husband, we realized that the Bluetooth device was too complicated, (there were) too many steps, so we said we'd put the music on the TV, so we adapted the tool, first adapting the environment to make it as simple as possible, and then that, well those are the strategies” (FG3, P3 – #436)

The third category, the *educational approach* focuses primarily on the adoption of appropriate communication, including popularized teaching, awareness-raising for the older adult and their loved ones, adapted demonstrations, and written and visual instructions. For example, a participant said:

“I try to find YouTube videos or things like that to **show** them. I try to have a demonstrator with me, like clocks, alarms, all that” (FG1, P6 – #316)

The use of these modalities enables a better understanding of how to use ATC and how to apply learning techniques at home. OTs also believe in the importance of paying particular attention to the vocabulary used, combining demonstrations with the use of videos to show how to use the ATC, and having a demonstration ATC close at hand.

Finally, the collaborative approach emphasizes involving other healthcare professionals and family caregivers in recommending an ATC. Engaging care staff and family members creates a support network that aids in implementing, learning, and integrating ATC use at home. A family member can practice with the individual and reinforce learning over time, enhancing the intervention's sustainability. OTs note that a proactive and well-prepared family presence significantly facilitates the application of learning techniques. On this subject, one participant said:

“I try not to be alone with the person if I have any doubts about the fact that he or she will be able to apply my techniques, so that was a factor that made the sustainability of my interventions a little easier” (FG2, P3 - #352).

## Discussion

The study is one of the first descriptive qualitative studies to identify factors influencing OTs’ decisions to prescribe ATCs for older adults with cognitive impairments in home settings. Our findings indicate that (1) various person-, activity-, technology-, and context-related conditions impact OTs’ decisions regarding ATC recommendations, and (2) multiple intervention and treatment approaches are essential for the effective use of any ATC.

Several factors were found to influence OTs’ decision to prescribe ATCs. First, in terms of human dimensions, the effectiveness of ATC interventions is thought to be affected by older adults’ life expectancy, cognitive abilities, motivation, and technological experience. Studies have indeed shown that residual cognitive abilities, and in particular learning abilities, facilitate the adoption and mastery of ATCs (Imbeault et al., 2018a; Imbeault et al., 2018b; Kenigsberg et al., 2019; Kerkhof et al., 2022). In our study, OTs specified that older adults with preserved procedural memory could more readily benefit from an ATC intervention. Procedural memory is a type of implicit memory that is preserved in Alzheimer's disease and mild cognitive impairment and allows the person to perform learned tasks automatically, without conscious thought (De Wit et al., 2021, 2023). This preservation forms the foundation of the errorless learning method, which minimizes errors through guided repetition (De Werd et al., 2015; Provencher et al., 2008; Voigt-Radloff et al., 2017). Our findings suggest that while OTs recognize the value of this approach in enhancing ATC adoption, safety, and independence for individuals with cognitive impairments, they often face contextual constraints that limit its application, particularly in cases with low rehabilitation potential. Our study underscores the need to promote early access to OT interventions along the continuum of neurocognitive disorders to maximize the potential of ATCs in improving safety, independence, and quality of life.

Other important cognitive abilities supporting the effective use of ATCs, and identified by OTs, were initiating tasks and perceiving errors; they were even considered as a prerequisite. It has been shown that a loss of these abilities is associated with daily functional deficits in the dementia continuum, which reinforces the need to develop ATCs designed to compensate for these abilities (McAlister et al., 2015; Schmitter-Edgecombe & Parsey, 2014). Indeed, home safety implicitly requires risk management involving initiating actions quickly, adapting to changes in routine, and reacting to unexpected events or correcting errors. These results align with other studies involving this population (Spalla et al., 2024), which suggest that planning is one of the most challenging task-related operations for older adults and requires significant assistance. Moreover, in our study, participants acknowledged their own limited advanced knowledge of ATCs, attributing this to a lack of training and peer knowledge-sharing. Limited interaction with ATC companies may also contribute to this knowledge gap. These results provide explanations for previous findings, as it was documented that only 12.4% of OTs across Canada use ATCs in their clinical practice (Aboujaoudé et al., 2021). Our results therefore highlight a critical need to both advance research on ATCs in occupational therapy and develop more advanced training programs for clinicians to enhance their knowledge and application of these technologies. Our findings also underscore those contextual conditions significantly shapes OTs decisions to recommend ATCs, a point well supported in the literature. For instance, participants described late referrals in homecare as limiting opportunities for ATC use—what one termed “fire extinguishing mode.” This mirrors a study that highlights how delayed interventions and limited follow-up reduce ATC effectiveness (Persson et al., 2020). Our results also showed that outpatient settings facilitate earlier, more proactive use of ATCs, in line with (Thawisuk et al., 2023). The presence of caregivers, identified in our study as a key condition for successful implementation, is similarly recognized in the literature as a facilitator of technology use (Thordardottir et al., 2019). Finally, barriers such as limited training, cost, and unequal access to technologies noted by our participants reflect findings from global OT surveys (Margot-Cattin et al., 2024). These parallels highlight the need to consider both individual and contextual factors in ATC-related clinical reasoning.

Second, OTs in the present study stressed the importance of personalized training and ongoing support as essential conditions for maximizing the benefits of ATCs, highlighting the importance of a tailored approach in intervention strategies, which aligns with the role of OTs when prescribing ATCs (O’Neill & Gillespie, 2008). According to participants, ATC prescriptions should be grounded in a comprehensive OT rehabilitation approach, combining gold-standard interventions like cognitive rehabilitation with classical OT approaches, including person-centred empowerment, educational, and collaborative methods. Our findings show that OTs regard cognitive rehabilitation as highly effective and believe these interventions should be applied when time and resources allow. This aligns with existing literature, which shows that errorless learning techniques can be effective in teaching skills necessary for everyday tasks (Provencher et al., 2008; Shiel et al., 1994; Voigt-Radloff et al., 2017). The primary objective of this method is to enhance the ability to learn specific, meaningful tasks, and evidence suggests that it is a gold standard (De Werd et al., 2015). Future studies should document how OTs incorporate this approach into practice and explore why, despite viewing it as the ideal standard, they do not consistently apply it due to limited time and resources. Among the other interventions that OTs use to support the prescription of ATC, there is the person-centered & empowerment approach which focuses on offering the person various alternatives in terms of the type of ATCs that can meet their needs, ensuring acceptability of the ATC by the person, and adapting assistive communication devices to individual needs (Townsend et al., 2007). The educational approach focuses on adapted communication through simplified teaching, awareness-raising for clients and families, written and visual instructions, and step-by-step demonstrations of ATCs. The collaborative approach includes other health professionals and family members. Together, these approaches support OT rehabilitation, incorporating ATC trials, scenarios, repetitions, and cognitive rehabilitation methods to ensure the effective use of technological aids by older adults. This is in line with what is considered standard cognitive rehabilitation clinical practice (Bahar-Fuchs et al., 2019; Gavelin et al., 2020; Kudlicka et al., 2019). In addition, it is documented that people with Alzheimer's disease may benefit from cognitive rehabilitation, such as errorless learning techniques to be able to use an ATC (Bier et al., 2006b, 2006a; Provencher et al., 2008).

Third, OT participants identified factors influencing ATC adoption related to activity meaningfulness, such as whether the ATC supports an active lifestyle, enables meaningful activities, prevents risks, and enhances occupational performance—factors consistent with existing AT literature (Lindqvist et al., 2013; Thawisuk et al., 2023). This reinforces the idea that ATCs fulfill the same needs to support the activity as other assistive technology, just as a cane supports the physical ability to walk, an ATC aids the cognitive ability to plan tasks and yet they are less recommended in clinical practice (Gagnon-Roy et al., 2024).

Finally, contextual conditions, such as the availability of caregivers, organizational support, and financial resources, play a crucial role in OTs’ recommendations and use of ATCs, as confirmed by previous studies (Persson et al., 2020). Time and resource constraints faced by OTs can limit the effectiveness of their interventions, including the prescription of ATCs. Indeed, in previous studies, OTs mentioned that organizational limitations and time constraints were barriers to ATC prescription (Persson et al., 2020). Our study adds a layer of precision to contextual factors’ influence on the success of this type of intervention, as participants suggested that limited OT knowledge and training in the field of cognitive impairments and ATCs, limited knowledge sharing and a limited presence of companies that provide ATCs around the practice highly influence ATCs prescriptions. These results support prior recommendations related to the integration of conferences and training programs for OTs on the latest ATCs to enhance their confidence and proficiency in prescribing ATCs. Our findings also highlight the significant impact of customer service and company branding on OTs’ choices of ATCs, an aspect well-known in marketing circles (Brodie et al., 2009) but previously undocumented specifically targeting OTs working in geriatrics. This highlights the importance for companies to develop marketing strategies that enhance their visibility among OTs, particularly through information sessions and direct solicitations. Providing comprehensive training and evidence-based resources can further support OTs in their practice, ultimately improving their interventions with their clients.

### Study Limitations

Focus groups have been shown to be effective for exploring clinicians’ perspectives on specific topics. The dynamic interaction in group discussions often fosters reasoning development, yielding insights that may not emerge in individual interviews. Although fewer than expected participants attended due to recruitment challenges (e.g., a national healthcare strike), the smaller groups allowed for careful listening, thought elaboration, and extensive responses, resulting in a rich data set. Moreover, the diversity in participants’ age and work experience, along with representation from various parts of the healthcare organization, supports the findings’ transferability for other OTs working with clients with cognitive impairments. Co-coding and co-validation by senior researchers and an independent co-researcher have reinforced the analysis credibility. Finally, data saturation was noted, reinforcing the credibility of the results obtained from the analysis.

## Conclusion

Our findings contribute to a deeper understanding of the factors influencing occupational therapists’ (OTs) decisions to prescribe assistive technology for cognition (ATCs). Key determinants include older adults’ life expectancy, cognitive abilities, motivation, and prior experience with technology. Additionally, our study reinforces the importance of anchoring ATC prescriptions within a comprehensive OT rehabilitation framework, integrating cognitive rehabilitation, empowerment, person-centered care, educational strategies, and collaborative approaches. This research advances knowledge by emphasizing the crucial role of OTs in ATC prescription, while also identifying barriers such as organizational limitations and lack of training**,** which hinder adherence to standard practices like cognitive rehabilitation. From a clinical perspective, our results highlight the practical challenges faced by OTs in prescribing ATCs. OTs primarily use ATCs to enhance safety and independence at home, especially when difficulties arise in complex tasks such as grocery shopping or medication management**.** However, barriers such as organizational constraints and limited knowledge necessitate stronger support from caregivers and ATC companies to ensure effective adoption. Notably, older adults without caregiver support are at higher risk of not receiving or fully benefiting from ATCs**.** To mitigate these disparities, ATC companies should provide additional support and after-sales services to facilitate OTs’ prescribing processes and reduce their workload.

## Key Messages

- OTs’ decision-making in ATC prescription is influenced by multiple factors related to the person, activity, and context. However, their ability to integrate ATCs effectively is often hindered by organizational barriers and limited advanced knowledge**.** Strengthening training in cognitive rehabilitation, person-centered empowerment, education, and caregiver collaboration is essential to optimizing ATC adoption in clinical practice.
- Successful ATC adoption depends not only on personal and technology factors but also on environmental support**.** The presence of family caregivers, sufficient financial resources, and strong post-prescription support from ATC providers play a crucial role. To bridge existing gaps in ATC access and effectiveness**,** more structured support from ATC companies and enhanced research on best prescribing practices are needed to ensure safety and independence for older adults at home.

## Disclosure Statement

JL is an innovation lab and product manager at Eugeria which distributes and designs ATCs. QDN is a medical expert at Eugeria.
